# Relationship between blood donors' iron status and their age, body mass index and donation frequency 

**DOI:** 10.1590/1516-3180.2013.1316554

**Published:** 2013-12-01

**Authors:** Ali Malekshahi Moghadam, Mahboobeh Mehrabani Natanzi, Mahmoud Djalali, Ahmad Saedisomeolia, Mohammad Hassan Javanbakht, Ali Akbar Saboor-Yaraghi, Mahnaz Zareei

**Affiliations:** I DVM. Researcher, Department of Nutrition and Biochemistry, School of Public Health, Tehran University of Medical Sciences, Tehran, Iran; II MSc. Doctoral Student, Department of Biochemistry, School of Medicine, Tehran University of Medical Sciences, Tehran, Iran; III PhD. Professor of Biochemistry, Department of Nutrition and Biochemistry, School of Public Health, Tehran University of Medical Sciences, Tehran, Iran; IV PhD. Assistant Professor, Department of Nutrition and Biochemistry, School of Public Health, Tehran University of Medical Sciences, Tehran, Iran; V MD, PhD. Researcher, Department of Nutrition and Biochemistry, School of Public Health, Tehran University of Medical Sciences, Tehran, Iran; VI PhD. Associate Professor, Department of Nutrition and Biochemistry, School of Public Health, Tehran University of Medical Sciences, Tehran, Iran; VII BSc. Laboratory Technician, Department of Nutrition and Biochemistry, School of Public Health, Tehran University of Medical Sciences, Tehran, Iran

**Keywords:** Blood donors, Iron, Ferritins, Male, Iran, Doadores de sangue, Ferro, Ferritinas, Masculino, Irã (Geográfico)

## Abstract

**CONTEXT AND OBJECTIVE:**

Regular blood donation may decrease body iron storage and lead to anemia. The aim here was to evaluate the iron status of Iranian male blood donors and the impact of age, body mass index (BMI) and donation frequency over one year, on iron status indices.

**DESIGN AND SETTING:**

Cross-sectional, descriptive and analytical study at Tehran Blood Transfusion Center, Tehran, Iran. METHODS: Between July and September 2011, 117 male blood donors were selected and divided into four groups according to their frequency of blood donation. Thirty male non-donors were also recruited as controls after adjusting for age, weight, height, smoking habits and monthly income. Iron status indices and some criteria such as general health and dietary measurements were determined among all subjects.

**RESULTS:**

The values of the iron-related parameters were significantly lower among donors than among non-donors. Only total iron binding capacity (TIBC) was found to be significantly higher among different donor groups than in the controls. A significant positive correlation was observed between age and serum ferritin (SF) only among the donors who had donated once within the preceding year. The iron status indices did not show any significant relationship with BMI among donors or non-donors.

**CONCLUSION:**

A donation frequency of more than twice a year had a significant influence on iron-related parameters. Therefore, without annual measurement of these parameters, further phlebotomies may lead to iron deficiency and donor rejection in the future.

## Abstract (pt)

**CONTEXTO E OBJETIVO:**

Doação de sangue regular pode diminuir o armazenamento de ferro no organismo, conduzindo à anemia. O objetivo foi avaliar o estado de ferro de iranianos doadores de sangue do sexo masculino e o impacto da idade, índice de massa corporal (IMC) e frequência da doação ao longo de um ano sobre os índices do estado de ferro.

**TIPO DE ESTUDO E LOCAL:**

Estudo transversal, descritivo e analítico no Centro de Transfusão de Sangue de Teerã.

**MÉTODOS:**

Entre julho e setembro de 2011, foram selecionados 117 homens doadores de sangue, divididos em quatro grupos de acordo com a frequência de doação. Trinta homens não doadores foram também recrutados como controles, após ajustes para idade, peso, altura, tabagismo e renda mensal. Índices do estado de ferro, bem como alguns critérios como a saúde geral e medidas dietéticas, foram determinados entre todos os indivíduos.

**RESULTADOS:**

Os valores dos parâmetros relacionados ao ferro foram significativamente mais baixos entre os doadores em comparação aos não doadores. Somente capacidade de ligação de ferro total foi significativamente maior entre os diferentes grupos de doadores em comparação com os controles. Correlação positiva significante entre idade e ferretina sérica foi observada somente nos que tinham doado uma vez no ano anterior. Os índices do estado do ferro não mostraram relação significativa com o IMC entre doadores e não doadores.

**CONCLUSÃO:**

A frequência de doação de mais de duas vezes por ano teve influência significativa nos parâmetros relacionados ao ferro. Assim, sem medidas anuais desses parâmetros, flebotomias adicionais podem levar a deficiência de ferro e rejeição de doadores no futuro.

## INTRODUCTION

Worldwide, more than 6.5% of people are blood donors.^1^ Blood donors are healthy subjects who donate their whole blood or blood components.^2^ Annually, large amounts of financial funding are expended on the safety of recipients, but very little attention is given to the state of wellbeing of blood donors.^3^ The overall occurrence of adverse events attributable to blood donation is 1%.^4^

Generally, investigations on donor care have highlighted immediate complications and body iron balance.^4^ Iron balance in donors is an influential safety issue.^5^ One complete blood donation (400-500 ml) eliminates almost 250 mg of iron. This corresponds to depletion of 4 to 10% of the entire body iron stores. Loss of red blood cells and plasma dilution result in diminution of hemoglobin (Hb) levels shortly after donation, by 0.5-1.0 g/dl.^6^ With continuous depletion of iron stores, the body adapts to reduced concentrations of iron or develops iron deficiency and anemia.^7^

Badar et al. showed that the prevalence of iron deficiency was 15-50% in men who had donated blood five to seven times within the preceding two years.^3^ Likewise, men who had donated three units of blood per year had a high potential risk of iron deficiency.^8^ Yousefinejad et al. showed that iron deficiency and iron deficiency anemia had a significant relationship with the number of donations per year and their intervals.^9^ Iron deficiency may be correlated with different observable health complications such as fatigue, low physical tolerance, impaired perception and restless leg syndrome (RLS).^10^ Additionally, iron deficiency leads to anemia and hence rejection of donors in the future.^11^ Among first-time donors, one of the most important reasons for not returning to donate is lack of qualification for donation.^12^ An appraisal of blood donation in Iran showed that just 34.8% of Iranian blood donors who had been rejected in 1999 gave blood over the subsequent three years.^13^

In the Iranian Blood Transfusion Organization (IBTO), Hb and/or hematocrit (Hct) assessments are used as a screening criterion for eligibility to donate blood. Since Hb concentrations may be normal even when iron stores are diminished, such individuals are possibly in danger of increasing iron deficiency anemia.^14^ Furthermore, iron status is influenced more by the frequency of donation over a one-year period than by the number of lifespan donations.^14^ ^,^ ^15^ On the other hand, it has been shown that there is high variation of the concentration of iron status parameters among females due to menstrual blood loss or pregnancies.^7^

## OBJECTIVE

This study was undertaken to determine the iron status of Iranian male blood donors and the influence of age, body mass index (BMI) and donation frequency per year on iron-related markers.

## METHODS

This cross-sectional, descriptive and analytical study was conducted at Tehran Blood Transfusion Center to assay the iron status of male blood donors. The study protocol was approved by the Ethics Committee of Tehran University of Medical Sciences, Tehran, Iran. Additionally, written informed consent was obtained from all participants prior to their entry into the study. 

### Subjects

One hundred and twenty donors who attended in Tehran Blood Transfusion Center from July to September 2011 were selected and enrolled to the study, according to the inclusion criteria. Subjects were randomly allocated to each group, using random number generation (RNG) of a computer. Finally, 117 volunteers (aged from 30-60) fulfilled the study protocol. 

The standard instructions used by IBTO to screen donors were followed in order to recognize eligible subjects. The minimum Hb cutoff for blood donation was 12.5 g/dl, and the general IBTO guidelines require a minimum interval of two months between donations, with a maximum of four and three donations a year allowed for men and women, respectively. The other constituents of the inclusion criteria in the study included a minimum weight of 50 kg; systolic and diastolic blood pressures in the ranges of 100-180 mmHg and 50-100 mmHg, respectively; heart rate between 50 and 100 bpm; body temperature no more than 37.5 °C; no history of donation outside of the IBTO; no major surgery in the preceding six months; no history of minor surgery within the previous 72 hours; no record of nutritional supplement intake or use of medications known to influence iron status within the last three months; no addiction to drugs and/or alcohol; no history of tattooing during the previous six months; no current communicable or non communicable diseases, especially cardiovascular disease (CVD), diabetes or polycythemia vera; and no record of vaccination during the previous four weeks. 

A total of 117 male blood donors and 30 healthy male non-donors who were matched for age, weight, height, smoking habits and monthly income were enrolled in the study. 

According to the frequency of donations within the previous year, the male blood donors were divided into four groups: group Ι (one donation), group II (two donations), group III (three donations) and group IV (four donations) with 27, 30, 30 and 30 people in each group, respectively. 

### General health assessment

Data on general health was collected by means of interviews and completion of a comprehensive questionnaire on age, height, weight, BMI [weight (kg)/height (m)^2^], heart rate, blood pressure, body temperature, smoking habits, past medical history, medications, frequency of donations per year, reason for phlebotomy, interval from the previous donation and some general data comprising literacy, occupation, monthly income, marital status and family size. 

### Dietary assessment

The volunteers completed a food frequency questionnaire (FFQ) and 24-hour dietary recall to establish their dietary intake. Consequently, daily energy and nutrient intakes were assessed using a nutritional software package (Food Processor II Windows v. 7.6; ESHA Research, Salem, Oregon, United States). 

### Laboratory tests

Prior to donation, ten milliliters of venous blood was drawn from each volunteer in non-fasting state and divided into ethylene-diaminetetraacetic acid (EDTA) and non-EDTA tubes. Uncoagulated blood was used for measurement of hematological parameters [Hb, Hct and mean corpuscular Hb concentration (MCHC)], using an automated cell counter (K1000, Sysmex, Kobe, Japan). The clotted sample was stored at room temperature for a maximum of one hour and then was centrifuged at 2500 g for 20 minutes. Afterwards, the serum was extracted and transferred to another tube for biochemical analysis. Serum iron (SI) and total iron binding capacity (TIBC) values were measured by means of a colorimetric method using approved diagnostic kits (Pars Azmun, Tehran, Iran). Transferrin saturation (TS) was calculated as the ratio of SI (µg/dl)/TIBC (µg/dl) × 100. Serum ferritin (SF) concentration was determined using a radioimmunoassay (RIA) (Immunotech Acoulter, France). All laboratory tests were undertaken in the laboratory of the Central Headquarters of Tehran Blood Transfusion Center. 

### Statistical analysis

The results were presented as mean ± standard deviation (SD). Data were put through one-way analysis of variance (ANOVA) and the Kruskal-Wallis H test. Then, if justified by the resulting statistical probability (i.e. P ≤ 0.05), Tukey's HSD test was used. Additionally, Pearson's correlation coefficient was also applied where required. Differences throughout this study were regarded as significant if P < 0.05. All data were analyzed using the Statistical Package for the Social Sciences (SPSS) 16.0 software (SPSS Inc., Chicago, Illinois, United States).

## RESULTS

As shown in Table 1, there were no significant differences in age, height, weight, BMI, smoking habits or monthly income among the study participants. The daily intake of macro and micronutrients, including energy, protein, fiber, iron, vitamin C, vitamin B12, folic acid, vitamin A and vitamin E, did not statistically differ among the various groups of volunteers (Table 2). Based on FFQ analysis, the most significant sources of dietary iron were meat, poultry and fish. Interestingly, both the blood donors and the non-donors presented only infrequent to moderate consumption of iron absorption inhibitory factors, such as dairy products, cereals, legumes, coffee and tea (data not presented). High daily intake of vitamin C (as a promoter of iron absorption) was also found.

**Table 1:** Selected characteristics of study participants according to annual blood donation frequency*,†

Variable	Non-donors (n = 30)	Blood donors (n = 117)			
		1 time (n = 27)	2 times (n = 30)	3 times (n = 30)	4 times (n = 30)
Age (years)	42.10 ± 8.06	43.62 ± 7.98	42.00 ± 7.64	42.27 ± 10.52	39.93 ± 7.77
Height (cm)	172 ± 0.05	173 ± 0.05	174 ± 0.05	174 ± 0.05	173 ± 0.05
Weight (kg)	82.17 ± 10.39	82.89 ± 10.82	86.20 ± 13.67	86.10 ± 14.23	2.90 ± 13.05
BMI (kg/m2)	27.41 ± 2.36	27.47 ± 2.05	28.28 ± 3.04	28.25 ± 3.25	27.42 ± 2.92
Smoking habits‡ (number/day)	1.37 ± 4.17	0.85 ± 2.58	0.23 ± 1.27	1.63 ± 4.95	0.0 ± 0.0
Monthly income‡ (USD)	520 ± 724	565 ± 535	400 ± 263	360 ± 234	565 ± 720

BMI = body mass index

* Values are expressed as mean ± standard deviation

† No statistically significant differences between donors and non-donors for P < 0.05

‡ Since the results relating to smoking habits and monthly income were not normally distributed, despite use of analysis of variance (ANOVA), the findings were expressed using the Kruskal-Wallis H test.

**Table 2:** Daily energy and nutrient intakes of subjects*,†

Daily intake	Non-donors (n = 30)	Blood donors (n = 117)			
		1 time (n = 27)	2 times (n = 30)	3 times (n = 30)	4 times (n = 30)
Energy (kcal)	1383 ± 111	1393 ± 98	1404 ± 102	1396 ± 111	1378 ± 118
Protein (g)	43.17 ± 3.33	42.90 ± 2.78	44.10 ± 2.07	43.52 ± 2.03	44.36 ± 2.64
Fiber (g)	13.79 ± 1.76	13.90 ± 1.41	13.85 ± 1.39	13.62 ± 1.27	13.80 ± 1.29
Iron (mg)	7.50 ± 0.95	7.31 ± 1.09	7.23 ± 1.03	6.93 ± 1.07	7.02 ± 1.11
Vitamin C (mg)	100 ± 19	100 ± 17	98 ± 16	94 ± 18	95 ± 13
Vitamin B12 (μg)	2.00 ± 0.30	1.89 ± 0.32	1.86 ± 0.32	1.85 ± 0.37	1.76 ± 0.34
Folic acid (μg)	177 ± 39	180 ± 32	166 ± 39	170 ± 35	170 ± 31
Vitamin A (μg)	986 ± 223	1008 ± 154	962 ± 149	931 ± 167	906 ± 155
Vitamin E (mg)	6.07 ± 1.35	6.23 ± 1.24	6.58 ± 1.10	6.18 ± 1.07	5.84 ± 1.18

* Values are expressed as mean ± standard deviation

† No difference (P > 0.05) was found between donors and non-donors.

As shown in Table 3, the values of Hb, Hct, MCHC, SF, SI and TS appeared to be significantly lower in the male blood donors than in the non-donors (P < 0.05). Only the TIBC values were found to be significantly higher among various donor groups, compared with non-donors (P = 0.03). 

**Table 3:** Laboratory indicators of iron status in donors and non-donors*

Variable	Non-donors (n = 30)	Blood donors (n = 117)	P-value
Hemoglobin (g/dl)	15.65 ± 1.12	14.65 ± 1.58	P = 0.001
Hematocrit (%)	44.89 ± 3.35	43.22 ± 3.72	P = 0.02
MCHC (%)	34.89 ± 1.20	33.85 ± 1.64	P = 0.001
Serum ferritin† (ng/ml)	118.89 ± 70.84	53.95 ± 53.98	P < 0.001
Serum iron (µg/dl)	127.21 ± 38.57	111.36 ± 35.02	P = 0.03
TIBC (µg/dl)	320.17 ± 56.09	339.30 ± 41.01	P = 0.03
Transferrin saturation (%)	40.76 ± 8.15	34.11 ± 12.51	P = 0.006

MCHC = mean corpuscular hemoglobin concentration

TIBC = total iron binding capacity

* Values are expressed as mean ± standard deviation

† Since the serum ferritin levels were not normally distributed, despite use of analysis of variance (ANOVA), the findings were expressed using the Kruskal-Wallis H test.

Table 4 shows the distribution of iron status variables among various groups of donors and non-donors based on the frequency of blood donation per year. As shown in this table, there was a statistically significant difference in Hb levels between non-donors and subjects who had donated three whole blood units (group III; P = 0.007) or four whole blood units (group IV; P = 0.0001). Likewise, the difference in Hb levels between donors who had given blood once (group I; P = 0.001) or twice (group II; P = 0.008) and group IV was considered to be statistically significant. The mean for Hct among the subjects with four donations differed significantly from non-donors and from group I (P = 0.006 and P = 0.023, respectively). Overall comparisons of subjects without any history of phlebotomy within the preceding year, with groups III or IV, showed significant differences in MCHC levels (P = 0.001 or P = 0.0001, respectively). The difference in MCHC levels between donors who had given blood three times (P = 0.040) or four times (P = 0.002) and group I was considered to be statistically significant. Similarly, MCHC concentrations were significantly lower among the donors in group IV than among those in group II (P = 0.003). There was a significant difference in SF levels between non-donors and subjects who had donated two, three or four whole blood units (P = 0.0001, P = 0.0001 or P = 0.0001, respectively). A significant difference in SF concentrations between group I and subjects who had donated three (P = 0.010) or four (P = 0.022) units of blood per year was also observed. There was a significant difference in SI levels between those who had donated three (P = 0.035) or four (P = 0.029) whole blood units per year and non-donors. Likewise, statistically significant differences in SI values were observed between group I and donors donating three (P = 0.012) or four times (P = 0.009) in the last year. The difference in TIBC values between non-donors and group III or IV was considered to be statistically significant (P = 0.02 or P = 0.01, respectively). In a similar manner, statistically significant differences in TIBC values were observed for group I versus group III (P = 0.01) or group IV (P = 0.008). TS values were found to be significantly higher among the non-donors than in groups III or IV (P = 0.0001 or P = 0.0001, respectively). There was also a significant difference in TS values between those who had given blood once in the previous year and groups III or IV (P = 0.004 or P = 0.002, respectively).

**Table 4:** Iron-related parameters based on number of blood donations per year*

Variable	Non-donors (n = 30)	Blood donors (n = 117)				P-value
		Group I (n = 27)	Group II (n = 30)	Group III (n = 30)	Group IV (n = 30)	
Hemoglobin (g/dl)	15.65 ± 1.12	15.37 ± 0.94	15.06 ± 1.43	14.40 ± 1.65	13.82 ± 1.75	P < 0.001
Hematocrit (%)	44.89 ± 3.35	44.56 ± 2.70	43.68 ± 3.54	43.04 ± 4.17	41.71 ± 3.83	P = 0.006
MCHC (%)	34.89 ± 1.20	34.51 ± 0.95	34.46 ± 1.43	33.41 ± 1.40	33.06 ± 2.09	P < 0.001
Serum ferritin† (ng/ml)	118.89 ± 70.84	87.70 ± 89.2	53.5 ± 31.2	45.9 ± 29.7	39.10 ± 25.9	P < 0.001
Serum iron (μg/dl)	127.21 ± 38.57	125.58 ± 30.50	115.23 ± 33.23	100.89 ± 33.74	99.13 ± 30.22	P = 0.001
TIBC (μg/dl)	320.17 ± 56.09	322.84 ± 34.68	331.60 ± 36.22	350.82 ± 48.42	351.96 ± 44.75	P = 0.01
Transferrin saturation (%)	40.76 ± 8.15	40.56 ± 13.00	35.65 ± 12.28	30.30 ± 13.01	29.96 ± 11.78	P < 0.001

MCHC = mean corpuscular hemoglobin concentration

TIBC = total iron binding capacity

* Values are expressed as mean ± standard deviation

† Since the serum ferritin levels were not normally distributed, despite use of analysis of variance (ANOVA), the findings were expressed using the Kruskal-Wallis H test.

Statistically significant correlations between age and SF were found only among the donors who had donated one whole blood unit in the last year (*r* = +0.39; P = 0.04). Other iron status indicators did not significantly correlate with age, either among donors or among non-donors. Hematological and biochemical variables did not show any significant association with BMI either among donors or among non-donors. The number of blood donations per year was also significantly and inversely related to the Hb (*r* = -0.40; P < 0.001), Hct (*r* = -0.28; P < 0.001), MCHC (*r* = -0.42; P < 0.001), SI (*r* = -0.30; P < 0.001), SF (*r* = -0.48; P < 0.001) and TS (*r* = -0.36; P < 0.001). On the other hand, it was directly correlated with TIBC (*r* = +0.32; P < 0.001). Additionally, SF concentrations were seen to have a significant positive relationship with the daily intakes of energy (*r* = +0.28; P = 0.04) and iron (*r* = +0.21; P = 0.03).

## DISCUSSION

Iron is a critically prominent substance in human metabolism. It plays a principal role in forming red blood cells and is also associated with many other cell functions in all the tissues of the body.^16^ Iron balance in blood donors is an influential safety issue.^5^ Indeed, blood donation can result in reduction of body iron stores.^7^ ^,^ ^17^

Considering that iron deficiency has been defined as SF concentrations < 12 ng/ml,^16^ no one in this study had iron deficiency according to SF levels. Although iron intake in the current study was lower than the Dietary Reference Intake (DRI) (around 7 mg/day rather than the recommended 8 mg/day), the subjects' diet was composed of low to moderate amounts of iron absorption inhibitory factors, high quantities of iron absorption enhancers and foods with highly bioavailable iron. This composition may be attributable to the high levels of literacy among the participants and, if it can be assumed that daily iron absorption progressively increases in donors,^18^ the diet explains the lack of iron deficiency among these subjects. Furthermore, the possibility that the participants might underreport their dietary macro and micronutrient intakes may be important.

As previously noted, the values of Hb (*r* = -0.40; P < 0.001), Hct (*r* = -0.28; P < 0.001), MCHC (*r* = -0.42; P < 0.001), SI (*r* = -0.30; P < 0.001), SF (*r* = -0.48; P < 0.001) and TS (*r* = -0.36; P < 0.001) were significantly and inversely correlated with donation frequency per year among the study participants. However, the TIBC values were directly related to annual donation frequency (*r* = +0.32; P < 0.001), thus showing that greater frequency of blood donation was accompanied by lower iron status. The findings from some other studies were somewhat similar to our results. Yousefinejad et al. found that Hb, SF, TIBC, SI and TS were significantly associated with frequency of donation.^9^ Likewise, many investigations have evaluated the impact of blood donation frequency per year on SF levels, and have reported that ferritin concentrations diminished markedly with increased blood donations.^8^ ^,^ ^19^ ^-^ ^22^

In contrast to our findings regarding Hb levels, no correlation was observed between levels of Hb and donation frequency in male blood donors in some other studies.^15^ ^,^ ^20^ ^,^ ^21^ ^,^ ^23^ The difference between the present study and these other relevant investigations may be explained by differences in the baseline Hb values, sensitivity and specificity of laboratory measurements, minimum donation interval, age range, food patterns and ethnic types of the participants in these surveys. Unlike our study, it has also been shown that indicators of iron status other than SF do not present contrasts between frequent and infrequent blood donors.^22^

In our study, the minimum Hb cutoff for blood donation was 12.5 g/dl. On the other hand, in some investigations, the minimum Hb value for male donors was ≥13.5 g/dl. Our findings about the relationship between Hb levels and the number of donations were consistent with the results of Norashikin et al. and Semmelrock et al.^5^ ^,^ ^24^ However, Punnonen et al. found that the Hb levels of multi-time donors did not notably contrast with those of donors who were giving blood for the first time.^25^ Likewise, concordant with our results, other studies found that the SF levels in male donors had a negative significant relationship with the number of donations.^5^ ^,^ ^26^

In the present study, there was a significant positive relationship between age with SF levels among donors who had donated once in the previous year (*r* = 0.39; P = 0.04). No significant correlation was observed between age and other iron status indices, either among donors or among non-donors. Similarly, in the investigation by Mittal et al., among individuals who had given blood once in the preceding year, the younger male donors (< 30 years of age) had lower SF concentrations than those over 50 years of age (P < 0.003).^14^ However, in another study, unlike our results, no relationship was observed between SF and the age of the male blood donors.^15^ Despite the similarity of some issues between the latter study and our investigation, the observed variance may be induced by differences in age range, laboratory assessments, baseline SF values, sample size, inflammatory status, dietary iron intake and absorption and possible multivitamin mineral supplement intake, as shown in the appraisal by Milman and Sondergaard (1984).^15^ Furthermore, although alcohol intake is not common in Islamic countries, it should not be neglected. Indeed, regardless of age and gender, the most important parameters influencing SF are blood donation, followed by alcohol intake in men.^27^

In the present study, there was no significant relationship between BMI and iron status indices among either the donors or the non-donors. In fact, with increasing BMI, iron status variables except for SF declined among both the donors and the non-donors, but these decreases were not statistically significant. On the contrary, in the study by Milman, significant positive interactions were found between SF and BMI among both the male blood donors and the controls (*r* = 0.17; P = 0.0001).^19^

However, considering the significant relationship between iron status markers and frequency of blood donation in the present study, it is essential to pay attention to certain safety issues, so as to ensure the donor's health and additional successful donations. Post-donation iron supplementation is one of the most common approaches for dealing with the complication of iron deficiency in blood donors.^28^ Iron supplementation not only elevates the iron stores in the volunteer donors, but also enables further blood donation in the future.^28^ The additional possible solutions for preventing iron deficiency in blood donors may include education, lifestyle modifications in relation to dietary patterns (thereby incorporating iron-rich foods) and assessment of iron store parameters such as SF, at least among multi-time blood donors.^29^ However, the notion of screening for iron deficiency among blood donors is debatable, principally because there is some antagonism between the probable advantages of iron deficiency associated with heart disease and the impacts that result in anemia.^30^

There are some limitations to the present study. Factors such as different ethnicities within Iran, dietary iron absorption and new indicators of iron status, including the Hb content in reticulocytes (CHr), the fraction of hypochromated red blood cells and the soluble transferrin receptor in serum (sTfR) were not taken into consideration in this appraisal. Prospective epidemiological studies and controlled clinical trials need to be implemented in order to register complete complications from blood donations. Subsequent studies in Iran should follow these lines. We also recommend that the present study should be expanded with larger sample sizes and longer follow-up time.

## CONCLUSION

Our findings showed that although none of the blood donors had iron deficiency, high annual donation frequency among males (more than twice a year) considerably influenced the iron status indices. Hence, if annual measurements of iron-related parameters such as SF are not made at least among regular blood donors, iron deficiency or iron deficiency anemia and subsequent loss of precious blood supply is unavoidable.
